# Cancer Progression Mediated by CAFs Relating to HCC and Identification of Genetic Characteristics Influencing Prognosis

**DOI:** 10.1155/2022/2495361

**Published:** 2022-10-15

**Authors:** Li Song, Qiankun Li, Yao Lu, Xianqi Feng, Rungong Yang, Shouguo Wang

**Affiliations:** ^1^Academy of Advanced Interdisciplinary Studies, Qilu University of Technology (Shandong Academy of Sciences), Jinan, Shandong Province 250353, China; ^2^Department of Tissue Repair and Regeneration, The First Medical Center of Chinese PLA General Hospital, Beijing, Beijing 250353, China

## Abstract

**Background:**

Hepatocellular carcinoma (HCC) is one of the most common malignancies, and although there are several treatment options, the overall results are not satisfactory. Cancer-associated fibroblasts (CAFs) can promote cancer progression through various mechanisms.

**Methods:**

HCC-associated mRNA data were sourced from The Cancer Genome Atlas database (TCGA) and International Cancer Genome Consortium (ICGC) database. First, the differentially expressed CAF-related genes (CAF-DEGs) were acquired by difference analysis and weighted gene coexpression network analysis (WGCNA). Moreover, a CAF-related risk model was built by Cox analysis. Kaplan-Meier (K-M) curves and receiver operating characteristic (ROC) curves were utilized to evaluate the validity of this risk model. Furthermore, enrichment analysis of differentially expressed genes (DEGs) between the high- and low-risk groups was executed to explore the functions relevant to the risk model. Furthermore, this study compared the differences in immune infiltration, immunotherapy, and drug sensitivity between the high- and low-risk groups. Finally, we verified the mRNA expression levels of selected prognostic genes by quantitative real-time polymerase chain reaction (qRT-PCR).

**Results:**

107 CAF-DEGs were identified in the HCC samples, and five prognosis-related genes (*ACTA2*, *IGJ*, *CTHRC1*, *CXCL12*, and *LAMB1*) were obtained by Cox analysis and utilized to build a CAF-related risk model. K-M analysis illustrated a low survival in the high-risk group, and ROC curves revealed that the risk model could accurately predict the 1-, 3-, and 5-year overall survival (OS) of HCC patients. In addition, Cox analysis demonstrated that the risk score was an independent prognostic factor. Enrichment analysis illustrated that DEGs between the high- and low-risk groups were related to immune response, amino acid metabolism, and fatty acid metabolism. Furthermore, risk scores were correlated with the tumor microenvironment, CAF scores, and TIDE scores, and CAF-related marker genes were positively correlated with all five model genes. Notably, the risk model was relevant to the sensitivity of chemotherapy drugs. Finally, the results of qRT-PCR demonstrated that the expression levels of 5 model genes were in accordance with the analysis.

**Conclusion:**

A CAF-related risk model based on *ACTA2*, *IGJ*, *CTHRC1*, *CXCL12*, and *LAMB1* was built and could be utilized to predict the prognosis and treatment of HCC.

## 1. Introduction

Liver cancer is one of the commonest malignancies. In accordance with the Global Cancer Statistics 2020, liver cancer is the 6th for incidence and 3rd in mortality among malignancy-related deaths [1–3]. Secondary, liver cancer includes hepatocellular carcinoma (HCC) and intrahepatic cholangiocarcinoma (ICC), of which HCC accounts for about 75-85%. Although various options such as chemotherapy with sorafenib, surgical resection, and liver transplantation are applied in treating HCC, but there is still a poor overall prognosis, with an overall survival (OS) of 3-5% [4–6]. Therefore, it is essential to find available targets for HCC treatment [7].

Cancer-associated fibroblasts (CAFs) can secrete growth factors, cytokines, and inflammatory ligands, which stimulate epithelial-mesenchymal transformation (EMT), promote tumor proliferation and migration, and induce therapy resistance and immune exclusion [8–10]. Studies showed that CAFs engaged in bidirectional signaling with liver progenitor cells and can act as cancer stem cells, suggesting a close link between cirrhosis and liver cancer development [11]. In addition, CAFs support tumor growth in the liver. For example, CAFs can influence tumorigenesis by altering ECM stiffness. For example, CAFs can influence tumorigenesis by altering ECM stiffness; moreover, the cytokines and other factors secreted by CAFs may promote tumor growth, tumor angiogenesis, and epithelial to mesenchymal transition (EMT) [12].

In this study, samples in the TCGA dataset were grouped into high CAF/low CAF score groups with CAF scores, and then, 107 differentially expressed CAF-associated genes (CAF-DEGs) were utilized for risk regression analysis. Furthermore, 5 prognostic genes were gotten and utilized to establish a risk model, which provided a reference for applying CAF-associated genes (CAFGs) in the clinical prognosis and treatment outcome of HCC.

## 2. Materials and Methods

### 2.1. Data Source

The mRNA expression data of 50 normal and 371 HCC samples, of which 360 HCC samples have available survival data, were sourced from The Cancer Genome Atlas database (TCGA). The mRNA expression data of 243 HCC samples were acquired from the International Cancer Genome Consortium (ICGC) database as a validation set.

### 2.2. Evaluation of the CAF Status in HCC

xCell can calculate the abundance of various cells based on the single-sample gene set enrichment analysis (ssGSEA), which includes cancer-associated fibroblasts [13]. This study counted the mass of 21 immune cells in 421 samples of TCGA-HCC dataset by xCell. The samples were grouped into high and low CAF with the median number of CAF cells. Kaplan-Meier (K-M) survival analysis was performed based on the high and low CAF groups and the survival information of the HCC samples. Then, we collated the clinical traits of the samples, STAGE subgroups, and GRADE subgroups and compared the differences in the proportion of CAF cells between the STAGE subgroups and GRADE subgroups using chi-square tests.

### 2.3. Identification of CAFGs by Weighted Gene Co-expression Network Analysis (WGCNA)

The genes with similar expression patterns can be gathered, and the module that was highly correlated with traits can be filtered by WGCNA, thus finding the target genes relevant to the study [14]. To further identify CAFGs, we performed a WGCNA analysis. First, we clustered the 371 HCC samples to see the overall correlation of all samples in the dataset. The soft threshold was determined to ensure that the interaction between genes maximally conformed to the scale-free distribution, and then, the coefficient of dissimilarity between genes was introduced based on the adjacency between genes, and the systematic clustering tree between genes was obtained accordingly. Similar modules analyzed by the dynamic tree cutting algorithm were merged (MEDissThres = 0.2). Finally, the correlations between the modules and CAF were calculated, and the key modules were selected with the criteria of |cor| > 0.4, *p* < 0.05. Moreover, the genes in the key modules were the CAFGs.

### 2.4. Identification of CAF-DEGs

We performed a differential analysis in the TCGA dataset for high CAF samples and low CAF samples to obtain differentially expressed genes (DEGs) between high and low CAF samples and differential analysis for normal and HCC samples. The screening condition for the differential analysis was *p* adjust. < 0.05 and |log2FC| > 0.5. To identify CAF-DEGs, we crossed CAFGs, DEGs between high and low CAF, and DEGs between normal and HCC samples.

### 2.5. Construction and Validation of the Risk Model

In this study, 360 samples containing survival information in the TCGA dataset were grouped into a training set and a test set with 7 : 3 (252 : 108), and the data in the training set were utilized to establish the risk model; firstly, the genes were verified as risk factors by univariate Cox regression analysis. Then, the genes with *p* < 0.05 were used to construct the multivariate Cox regression model, using the stepwise regression function step, with the parameter direction set to both, to adjust the multivariate regression model, and the obtained genes were used as prognostic factors to build the model.

The risk value of each patient was counted by the expression of the genes, and the patients were grouped into high and low risk with the median risk value. Then, the risk profile was plotted and survival analysis for the high- and low-risk groups was conducted. In addition, we plotted the receiver operating characteristic (ROC) curve, and the area under curve (AUC) was used to indicate the prediction accuracy. Finally, the correlations between the risk model and clinical traits (age, gender, M, N, T, and other clinical data) were assessed using the chi-square test.

Next, we validated the risk model using the TCGA test set and the ICGC validation set. In these two datasets, cases were spanided into high and low risks, respectively, and risk profiles, survival curves, and ROC plots were plotted, and correlations between risk factors and clinical traits were analyzed.

### 2.6. Correlation of Risk Model and Clinical Traits

The clinical traits in the training set of TCGA-HCC data were collated, including age, sex, disease stage, T, N, and M. The samples were grouped according to the different clinical traits, and the risk values were compared between the different groups to see if there were significant differences and visualized by box plots.

#### 2.6.1. Independent Prognostic Analysis

The clinicopathological factors in the training set samples were added to the Cox analysis to investigate the independent prognosis of the risk model and clinicopathological factors. On this basis, a nomogram graph of the survival rate of the risk model and clinical factors was constructed. The factors that obtained significant results from the above multivariate Cox analysis were plotted, and the OS was predicted according to the total score. The correction curve was utilized to evaluate the prediction results of the model.

#### 2.6.2. Enrichment Analysis

We divided the TCGA dataset into the high- and low-risk groups. The samples in the high- and low-risk groups were analyzed for differences using the “limma” R package, and the log2|FC| were then sorted from highest to lowest. Gene Set Enrichment Analysis (GSEA) was conducted using the “clusterProfiler” R package to find the common functions and related pathways of a large number of genes in the differentially expressed gene set [15]. The thresholds set were |NES| > 1, NOM *p* < 0.05, and *q* < 0.25, and the databases used for GSEA were Kyoto Encyclopedia of Genes and Genomes (KEGG) and Gene Ontology (GO).

### 2.7. Correlation of Risk Score with Other Scores

To further validate the accuracy of the risk model in predicting CAF, we executed Spearman correlation analysis on the risk score, stroma score, immune score, ESTIMATE score, tumor score, the proportion of CAF predicted by xCell, and the proportion of CAF predicted by EPIC, MCP-counter, and Tumor Immune Dysfunction and Exclusion (TIDE). Firstly, the “ESTIMATE” R package was utilized for ESTIMATE analysis to obtain the immune score, stromal score, ESTIMATE score, and tumor score for each sample. The EPIC algorithm analyzed the percentage of infiltration of eight-cell types, including CAFs, based on expression data [16]. We used the MCP-counter to attribute the content of CAFs in the samples. The xCell algorithm can also predict the proportion of CAFs. Finally, the CAF content was obtained using TIDE. The correlations between risk scores and each index were calculated using the Spearman correlation analysis. *p* < 0.05 represents significant correlation.

### 2.8. Correlation between CAF Marker Genes and Prognostic Genes

There were 23 CAF-associated marker genes, including *ACTA2*, *ASPN*, *CAV1*, *COL11A1*, *COL1A1*, *COL1A2*, *COL3A1*, *EMILIN1*, *FAP*, *FN1*, *FOXF1*, *MFAP5*, *MMP11*, *MMP2*, *OGN*, *PDGFRA*, *PDGFRB*, *PDPN*, *S100A4*, *SLC16A4*, *SPARC*, *TNC*, and *ZEB1* [17, 18]. Then, we calculated the correlations between prognostic genes and risk scores with CAF marker genes.

### 2.9. Inferring Immune Cell Abundance in High- and Low- Risk Groups Using the ssGSEA Algorithm

ssGSEA is a single-sample GSEA method by which we can obtain the immune cell, of each sample [19]. Using 28 immune-related gene sets, we can get the immune activity. Then, the differences in 28 immune activities between the high- and low-risk groups were compared, and the differential immune activities were related to the risk scores.

### 2.10. Chemotherapy Drug Sensitivity Prediction

We know that the Genomics of Drug Sensitivity in Cancer (GDSC) database has many drug sensitivity and genomic datasets that are important for the discovery of potential oncology therapeutic targets. IC50 refers to the half amount of a drug that inhibits specific biological processes. The “pRRopheticPredict” R package (version 0.5) was utilized to calculate 138 drugs included in the database and compare differences in drug IC50 between the high- and low-risk groups.

### 2.11. Quantitative Real-Time Polymerase Chain Reaction (qRT-PCR) Validation

First, RNA was extracted from control cells WRL68 and HCC cells Huh7, Hepg2, and sk-sep-1, followed by a reverse transcription reaction, and finally, the target gene was amplified by PCR reaction. The RNA extraction kit was TRIzol Reagent (ref.: 15596018) kit provided by Ambion. The reverse transcription kit was the SweScript RT I First-strand cDNA Synthesis All-in-One^TM^ First-Strand cDNA Synthesis Kit (cat.: G33330-50) from Servicebio. PCR reactions were performed with the 2x Universal Blue SYBR Green qPCR Master Mix (cat.:G3326-05) kit from Servicebio. Primer sequences are shown in Table 1. The PCR reaction process was 95°C predenaturation for 1 min and then 40 cycles. Each cycle included 95°C denaturation for 20 s, 55°C annealing for 20 s, and 72°C extension for 30 s. The internal reference for gene detection is GAPDH. The expression of *ACTA2*, *IGJ*, *CTHRC1*, *CXCL12*, and *LAMB1* in normal cell WRL68 and HCC cells Huh7, Hepg2, and sk-sep-1 were compared by analysis of variance (ANOVA), and *p* < 0.05 was a difference.

## 3. Results

### 3.1. Evaluation of the CAF Status in HCC

**Figure 1:**
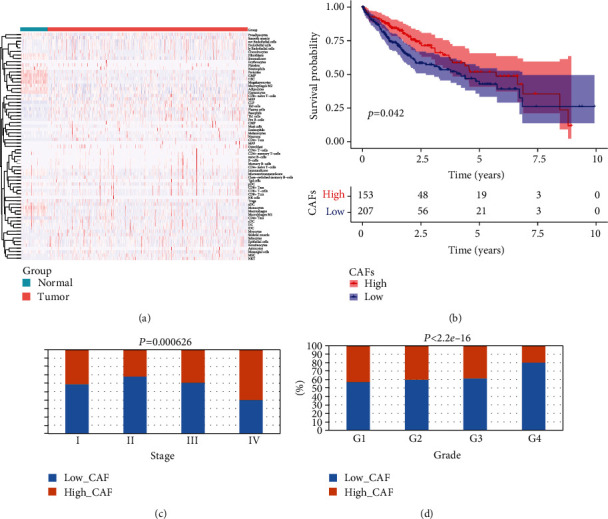
The changing trend of CAF in the TCGA-LIHC queue analyzed by the XCELL algorithm. (a) Heat map of different cell concentrations calculated by xCell. (b) K-M curve of high and low CAF group. (c) Correlation of CAF cells in different STAGE groups. (d) Correlation of CAF cells in different GRADE groups.

We calculated the immune cell content of 421 samples in the TCGA dataset (Figure 1(a)). After screening out the normal samples, there were 158 high CAF samples and 213 low CAF samples. The results of K-M analysis of the high and low CAF groups were shown (Figure 1(b)), and it can be seen that there was a significant survival difference between the high and low CAF groups. The results of clinical trait correlation between high and low CAF groups showed that CAF cells were different between different STAGE groups and between different GRADE groups (Figures 1(c) and 1(d)).

### 3.2. Identification of CAFGs by WGCNA Analysis

**Figure 2:**
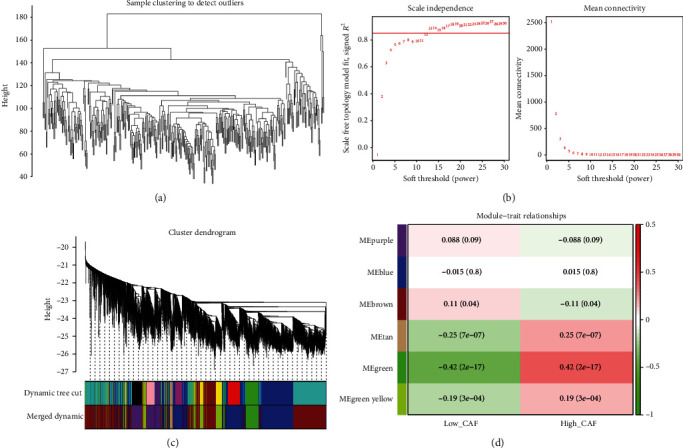
Identification of CAFGs by WGCNA analysis. (a) Sample clustering of TCGA dataset. (b) Scale-free soft threshold distribution. (c) Cluster tree of modules. (d) Heat map of correlation between modules and clinical traits. Each row corresponds to a module eigengene and each column to a trait. Each cell contains the corresponding correlation and *p* value. The table is color-coded by correlation according to the color legend.

The clustering of the samples in the TCGA dataset was shown in Figure 2(a), and the samples were not deleted. The power threshold was chosen as 13, so that the interactions between genes conformed to the scale-free network (Figure 2(b)). From the module clustering tree, we can see that 12 modules were clustered, and after merging, 6 modules were obtained (Figure 2(c)). Finally, the key modules were filtered according to their correlation with CAF, and we got the green module (Figure 2(d)). Therefore, 898 genes in the green module were used as CAFGs.

### 3.3. Identification of CAF-DEGs

**Figure 3:**
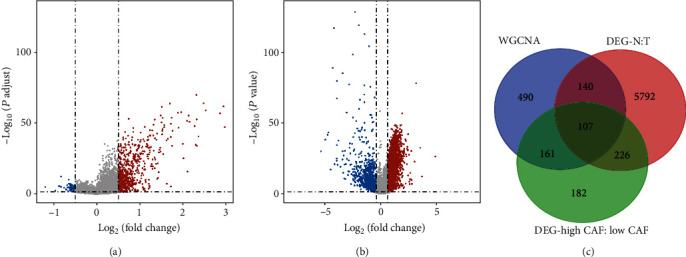
Identification of CAF-DEGs. (a) Volcano map of the gene from high CAF vs. low CAF samples. (b) Volcano map of the gene from HCC vs. the normal sample; (c) Venn diagram of CAFGs, DEGs between high and low CAF, and DEGs between normal and HCC samples. Red dots represent upregulation, and blue dots indicate downregulation.

There were 676 DEGs between the high and low CAF groups (Figure 3(a)). 6265 DEGs were found between normal and HCC samples (Figure 3(b)). CAFGs and DEGs between high and low CAF and DEGs between normal and HCC samples were crossed to obtain 107 CAF-DEGs, and the Venn diagram is shown (Figure 3(c), Table S1).

### 3.4. A Risk Model Based on 5 Genes Was Built

**Figure 4:**
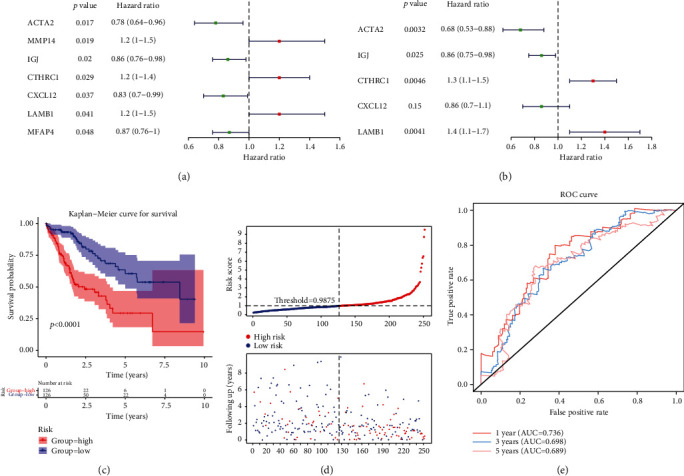
Identification of prognostic genes and evaluation of risk regression models. (a) Forest map of univariate Cox results. (b) Forest map with multivariate Cox results. (c) K-M survival curve of risk score. (d) Risk curves for the high- and low-risk groups. (e) The ROC curve evaluating the validity of the risk model.

**Figure 5:**
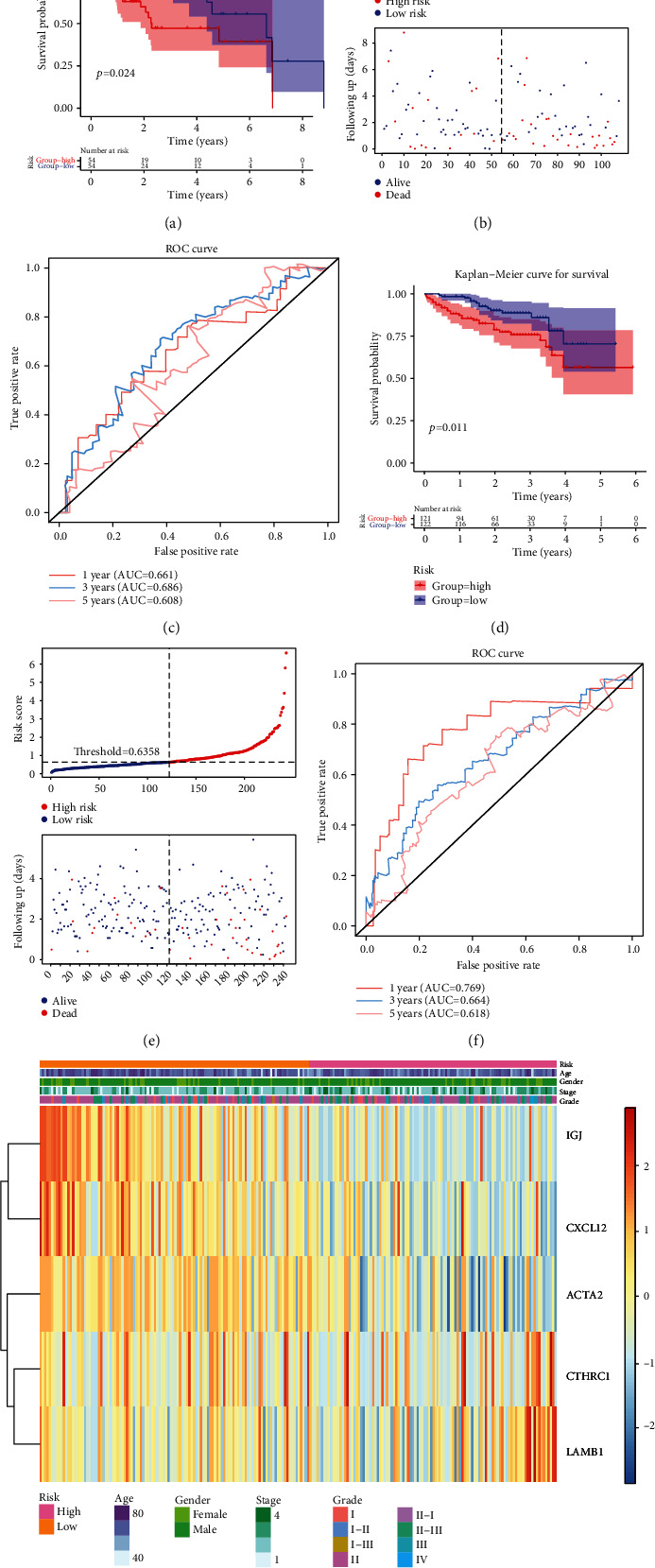
Testing and validation of the risk model. (a) K-M survival curve of risk score in the test set. (b) Risk curves for the high- and low-risk groups in the test set. (c) ROC curve in the test set evaluating the validity of the risk model. (d) K-M survival curve of risk score in the validation set. (e) Risk curves for the high- and low-risk groups in the validation set. (f) ROC curve in the validation set evaluating the validity of the risk model. (g) Overview of the correlation between risk score and clinical features in validation.

In the TCGA training set, univariate Cox analysis yielded 7 genes (Figure 4(a), Table 2). After multivariate Cox analysis, 5 genes appeared in multivariate Cox analysis (Figure 4(b), Table 3): *ACTA2*, *IGJ*, *CTHRC1*, *CXCL12*, and *LAMB1*. The risk value of each patient was counted from the expression of these five genes, and the cases were classified into high and low risks (median value = 0.988) (Figure 4(c)). The survival analysis of the high- and low-risk groups illustrated there was a significant survival difference between the high- and low-risk groups (Figure 4(d)). The AUC at 1, 3, and 5 years in the ROC curve were 0.661, 0.686, and 0.608, respectively (Figure 4(e)). In addition, in both the TCGA test set and ICGC validation set, the survival of the high-risk group was lower, and the AUC at 1, 3, and 5 years was more significant than 0.65 (Figures 5(a)–5(g)). In addition, in the ICGC validation set, grade was different between the high- and low-risk groups. It indicated that the risk model could be effectively used as a prognostic model.

### 3.5. Correlation of Risk Model and Clinical Traits

**Figure 6:**
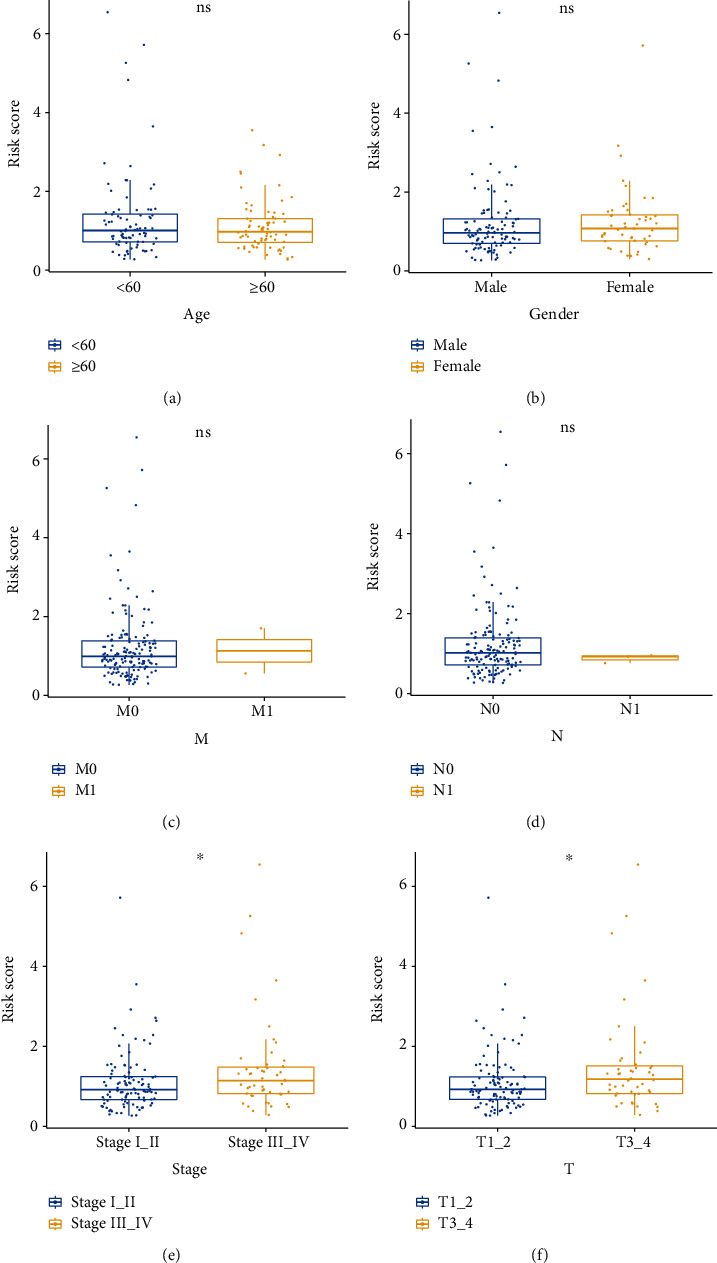
Correlation of risk model and clinical traits. (a) Correlation between risk models and age traits. (b) Correlation of risk models with gender. (c) Correlation between risk model and M traits. (d) Correlation between risk model and N traits. (e) Correlation between risk model and stage traits. (f) Correlation between risk model and T traits.

The correlation between the risk model and clinical traits showed that the risk values differed significantly between stages I-II and stages III-IV. And the risk values were quite different between *T*1 − 2 and *T*3 − 4 stages. The results were shown (Figures 6(a)–6(f)).

### 3.6. Risk Score and Stage Were Independent Prognostic Factors

**Figure 7:**
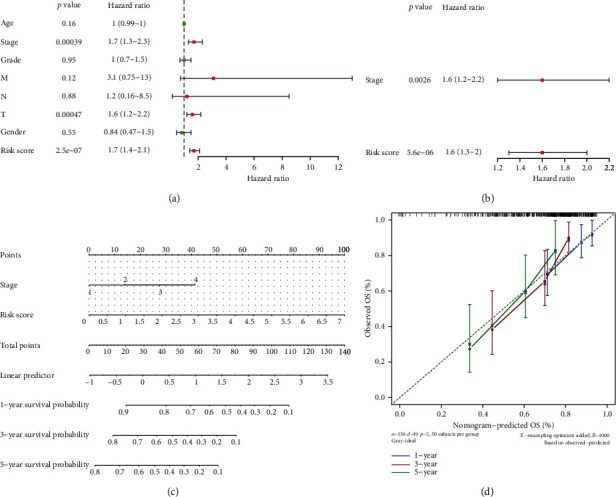
Risk model-independent prognosis in the training set. (a) Forest map of univariate Cox results. (b) Forest map with multivariate Cox results. (c) Survival nomogram graph. (d) Correction curve for line graph.

The factors with *p* < 0.05 in the univariate Cox regression analysis were T, risk score, and stage (Figure 7(a), Table 4). The three significant factors were added to the multivariate Cox analysis (Figure 7(b), Table 5), and the results showed that risk score and stage were significant. The survival nomogram graph was shown (Figure 7(c)). In the corrected curve, the *c*-index was 0.703, and the corrected *c*-index was 0.696, and the slopes were calculated to be 0.697, 0.406, and 0.300 at 1, 3, and 5 years, which demonstrated the best prediction at one year (Figure 7(d)).

### 3.7. Enrichment Analysis of High- and Low-Risk Groups

**Figure 8:**
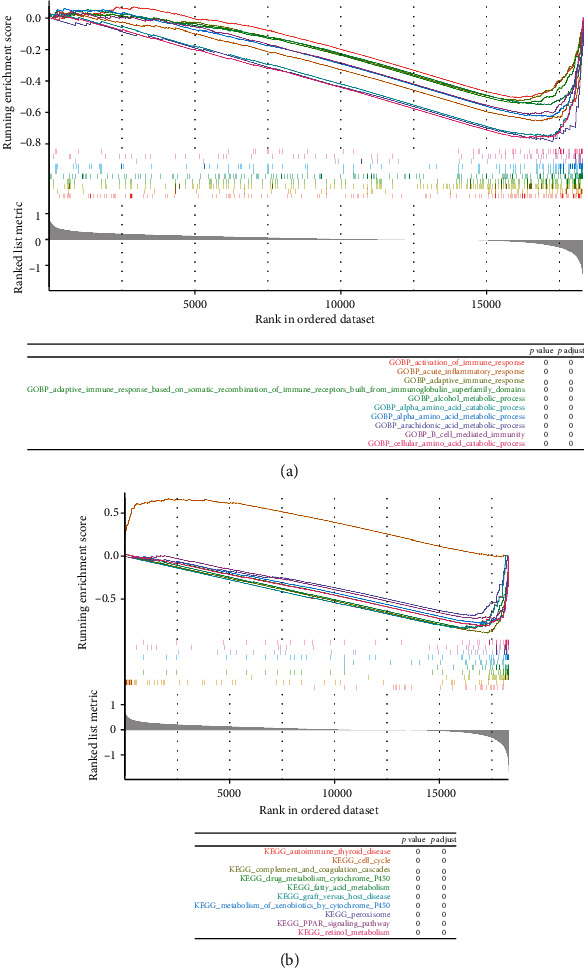
Enrichment analysis of the high- and low-risk groups. (a) The top10 KEGG pathways. (b) The top10 GO pathways.

A total of 73 KEGG paths and 1968 GO paths were enriched by GSEA, and we selected the top 10 KEGG paths and GO paths to visualize them. As can be seen (Figure 8(a)), the top 10 KEGG pathways obtained have activation of the immune response, alcohol metabolic process, alpha-amino acid metabolic process, and B cell-mediated immunity. The top 10 GO functions were autoimmune thyroid disease, cell cycle, graft versus host disease, peroxisome, PPAR signaling pathway, and retinol metabolism (Figure 8(b)).

### 3.8. Correlation of Risk Scores with Other Scores and Correlation of CAF Marker Genes with Prognostic Genes

**Figure 9:**
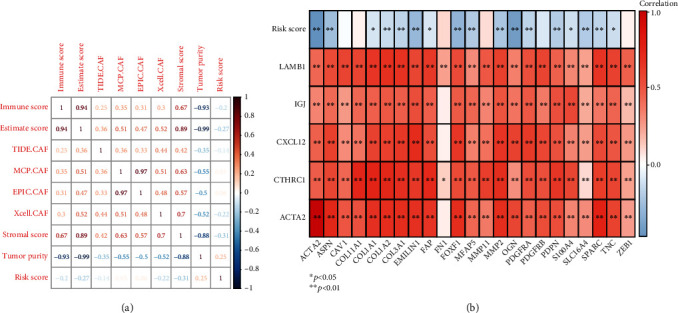
Correlation of risk scores with other scores and correlation of CAF marker genes with prognostic genes. (a) Heat map of correlations between risk score and other scores. (b) Heat map of correlations between CAF marker genes and prognostic genes.

The correlation results of the risk score with other scores suggested that the risk score was negatively relevant to the immune score, ESTIMATE score, stromal score, xCell-predicted CAF ratio, and TIDE-predicted CAF ratio, and positively relevant with the tumor score (Figure 9(a)). The correlations between prognostic genes and risk scores with CAF marker genes were calculated, and the results were as follows. The correlation results illustrated that risk scores were negatively related to*ACTA2*, *ASPN*, *COL1A1*, *COL1A2*, *COL3A1*, *EMILIN1*, *FAP*, *FOXF1*, *MFAP5*, *MMP2*, *OGN*, *PDGFRA*, *PDPN*, *S100A4*, *SLC16A4*, *SPARC*, and *TNC* genes. FN1 with LAMB1, CTHRC1, and SLC16A4 was positively associated with *ACTA2*, *IGJ*, *CXCL12*, and *LAMB1*. In addition, the remaining 21 CAF-related marker genes were positively associated with five prognostic genes (Figure 9(b)).

### 3.9. Inferring Immune Cell Abundance Using the ssGSEA Algorithm

**Figure 10:**
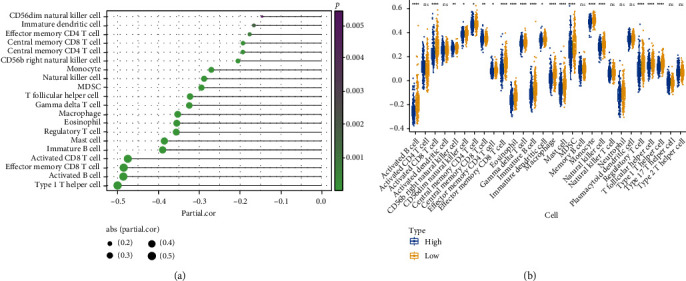
Inferring immune cell abundance in the high- and low-risk groups by the ssGSEA algorithm. (a) Correlation between cell contents and risk values. (b) Box plots of cell contents between the high- and low-risk groups.

As can be seen (Figure 10(b)), among the 28 cells, 20 cells were different between the high- and low-risk groups, including activated B cell, CD56bright natural killer (NK) cell, CD56dim NK cell, central memory CD4 T cell, central memory CD8 T cell, and Type 1 T helper cell, and the 20 significant cells were plotted separately from the risk score in a lollipop plot as follows (Figure 10(a)).

### 3.10. Chemotherapy Drug Sensitivity Prediction

**Figure 11:**
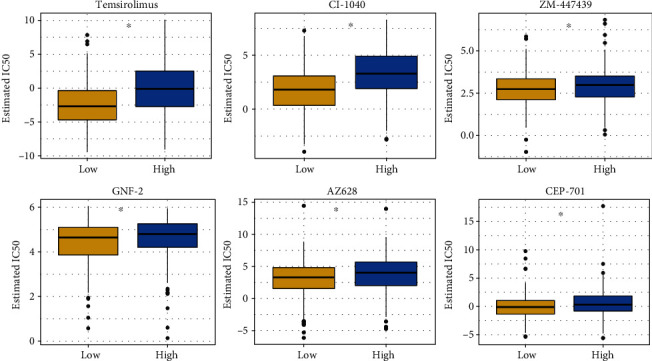
Significant differences of 6 drugs between the high- and low-risk groups.

According to the calculation results, 65 drugs showed differences in the high- and low-risk groups, which were temsirolimus, CI.1040, NU.7441, AZD8055, AICAR, AMG.706, DMOG, KU.55933, Metformin, EHT.1864, Dasatinib, NVP.BEZ235, PD.0325901, AZD.0530, NVP.TAE684, AKT.inhibitor.VIII, Vorinostat, GDC0941, PD.173074, Erlotinib, Docetaxel, WO2009093972, Rapamycin, AZD6244, JNJ.26854165, BI.D1870, MG.132, BX.795, A.770041, PD.0332991, Z.LLNle.CHO, AP.24534, Parthenolide, GW.441756, Nilotinib, OSI.906, X17.AAG, GDC.0449, AZD6482, WH.4.023, PF.4708671, Axitinib, TW.37, SB590885, Thapsigargin, NSC.87877, Cyclopamine, CMK, RDEA119, Gefitinib, Sorafenib, CEP.701, Imatinib, Methotrexate, ABT.263, Vinblastine, AZD7762, Lapatinib, AZ628, GNF.2, Bryostatin.1, Camptothecin, Nutlin.3a, FH535, and ZM.447439 (Table S2); they were visualized as a box plot as shown in the figure below.  Figure 11 showed box plots for just the six drugs in the high- and low-risk groups.

### 3.11. qPCR Validation

**Figure 12:**
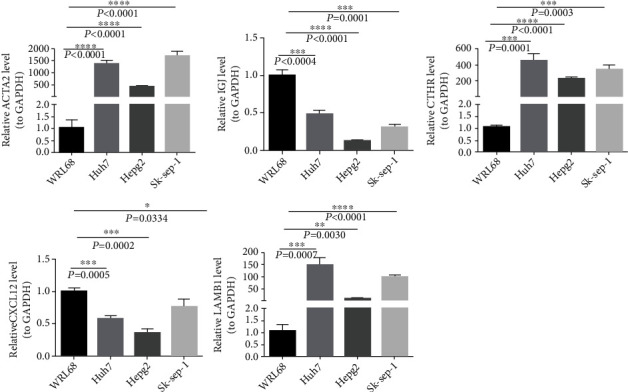
Validating the expression levels of the five genes in normal and HCC cells by RT-qPCR.

The results of qPCR demonstrated that expression levels of *ACTA2*, *IGJ*, *CTHRC1*, *CXCL12*, and *LAMB1* genes were different in normal cells WRL68 and HCC cells Huh7, Hepg2, and sk-sep-1. Specifically, *ACTA2*, *CTHRC1*, and *LAMB1* genes were significantly upregulated in HCC cells Huh7, Hepg2, sk-sep-1, and *IGJ*, *CXCL12* were downregulated in HCC cells (Figure 12).

## 4. Discussion

While there have been advances in diagnostic techniques and treatment of HCC, [20, 21] the survival prognosis remains poor because of its high recurrence and metastasis rates [22]. CAFs are the main cellular component that can affect the formation of liver fibrosis, which in turn results in the development of HCC [10, 12]. Many prognostic models for HCC have been presented by far. Zhang et al. built a prognostic model which was able to reasonably predict the prognosis of HCC patients and provided a new idea to study HCC of different histological grades [21]. Long et al. developed a four-gene prognostic model to probe the differences in mRNA expression between HCC and neighboring liver to obtain potential genetic biomarkers [2]. Wang et al. screened immune-related differentially expressed genes closely related to HCC and further detected genes associated with prognosis [23]. However, because of the limitations of the public database data, further validation of the proposed prediction models is necessary or regression modeling methods need to be applied to determine if the prediction accuracy can be further improved. More than that, the validity of the prediction model should be confirmed in a large sample of HCC. In this study, we sought five biomarkers basing CAFGs for a prognostic model for HCC by bioinformatics method, conducted an independent prognostic analysis and functional enrichment analysis, and calculated the differences between immunoassay (immune infiltration, immunotherapy) and drug sensitivity at all levels. At last, qRT-PCR verified the expression levels of *ACTA2*, *IGJ*, *CTHRC1*, *CXCL12*, and *LAMB1* genes in normal and HCC cells, which is a relatively complete work for the prognostic building.

In the present study, five genes have been obtained for the HCC prognostic model. *ACTA2*, actin alpha 2, which contributed to cell-generated mechanical tension and maintenance of cell shape and movement, was highly expressed in carcinomas [24]. Meanwhile, a previous study showed that CAFs enhanced the tumor-initiating and tumorigenic properties of HCC cells, and *ACTA2* was exactly a biomarker of CAFs. The upregulation of *ACTA2* level indicated poor survival HCC patients [25]. It was demonstrated that a linking chain of multisomal IgA and IgM is also present in *IGJ* [26]. It is possible that their upregulation may enhance the anticancer immune response to sorafenib treatment and facilitate the survival of HCC [27, 28]. In addition, overexpression of *CTHRC1* contributes to tumorigenesis and progression through positive regulation of tumor spread, invasion, migration, adhesion, and metastasis [29–31]. Immunohistochemical analysis demonstrated that CTHRC1 expression levels were elevated in HCC tissues [32]. Stromal-derived-factor-1 (*SDF-1*) was expressed in more than 23 different types and participated in tumor metastasis [33]. Interestingly, *SDF-1* protein for the HCC cells was expressed in the cytoplasm and nucleus [34]. Notably, the level of SDF-1 was lower in HCC. Patients with relatively high *SDF-1* showed longer OS [35]. *LAMB1* consists of laminins [36]. LamB1 mediated *β*1 integrin signaling and can regulate cell migration, proliferation, and survival by activating specific p67kDa laminin receptors (LamR) [37–39]. HCC patients have shown elevated levels of LamB1 in cirrhotic tissues, with further increased expression in HCC [40]. In HCC, the expression of the b1 integrin receptor and LamR were upregulated, which was relevant with enhanced tumor aggressiveness and poor patient survival [41, 42].

Based on the enrichment analysis of the high- and low-risk groups by GSEA, function ways of fatty acid metabolism, amino acid metabolism, and immune response were related to the progress of HCC seriously. Firstly, a specific reprogramming xiang of fatty acid metabolism has been found in the nonalcoholic steatohepatitis (NASH) stage of nonalcoholic fatty liver disease (NAFLD). The liver is involved in the context of MetS and simple steatosis can progress to liver fibrosis or even cirrhosis, and eventually to HCC [43]. Metabolic reprogramming can support hepatocyte proliferation by participating in fatty acid synthesis and oxidation [44]. Second, the synthesis of nonessential amino acids is vital for the maintenance of liver function [45, 46]. In HCC, abnormalities in amino acid and protein metabolism occur [47].

Tumor immune cells can be participated in the immune response to cancer and also predict treatment efficacy and survival [48]. In the current study, there were 20 immune cells that differed between the high- and low-risk groups, including B cells, T cells, and NK cells. Regulatory B (Breg) cells accumulate in the tumor environment, and it can produce high levels of IL-10. Breg can suppress the host immune responses to promote tumorigenesis in HCC [49]. Regulatory T cells (Tregs), expressing CD25 and forkhead boxP3 (FoxP3), were negative during immune surveillance, resulting in tumor tolerance [50]. There are fewer NK cells in HCC tissue and NK cells can inhibit cytokine production and cytotoxic activity [51]. Zhu et al. constructed the prognostic model and the recurrence risk model and found that patients with high risk scores responded strongly to immune checkpoint inhibitor therapy and that low-risk patients may derive more significant clinical benefit from chemotherapy [52].

65 drugs showed differences in the high- and low-risk groups. Temsirolimus is a prodrug of sirolimus. Studies have shown that temsirolimus has an inhibitory effect on HCC cells, and in phase I/II clinical trial, it was well-tolerated in HCC patients [53]. Moreover, temsirolimus is an mTOR inhibitor that can block cell cycle transition and affects cell proliferation by inhibiting mTOR and growth factors [54]. CI-1040, another drug predicted by our prognostic model, is an oral inhibitor of extracellular signal-regulated kinase (MEK) [55], It is a new candidate for targeted treatment of HCC because of its potential antitumor efficacy [56]. ZM447439 (ZM) induces apoptosis in HCC cells by interfering with spindle integrity and chromosome segregation [57]. These three drugs are representatives of anti-HCC drugs. However, among the 65 drugs, there are also some news, of which the effects on HCC are not definite. For example, GNF-2 inhibits the enzymatic and cellular kinase activities of ABL1, ABL2, and recombinant ABL and can inhibit the proliferation of fibroblasts. Still, its effect on anti-HCC have not been elucidated [58]. Then, AZ628, another new drug for HCC, can be involved in fibrosarcoma formation, and AstraZeneca can effectively inhibit cancer cell proliferation by inhibiting the activity of Raf [59]. CEP-701 can effectively inhibit trk receptors, leading to cell death in prostate cancer (PC), and it can also limit tissue penetration by binding serum proteins [60].

## 5. Conclusion

This study concentrated on the prognostic value of CAFs for HCC and identified CAF-related genes. A prognostic model of 5 CAFGs for HCC was developed in this research, and the expression of the five genes were verified by the qRT-PCR method. It provides new directions for the treatment of HCC. Nonetheless, one shortcoming of this study should be addressed, there are no clinical trials.

## Figures and Tables

**Table 1:** Primer sequences of genes used in qRT-PCR validation.

Gene	Forward	Reverse
GAPDH	CCCATCACCATCTTCCAGG	CATCACGCCACAGTTTCCC
ACTA2	CACAGAGCAAAAGAGGAATC	TCAGCAGTAGTAACGAAGGA
IGJ	CTCAAGAAGGTGAAAGGATT	TTTTTACAGAGGTCAGACAA
CTHRC1	AAGGAAGCCCTGAAATGAAT	CCACAGAAGAAGTGCGATGA
CXCL12	CACTCCAAACTGTGCCCTTC	CTTGTCTGTTGTTGTTCTTC
AMB1	GTTGTAAATCTTGTGCTTGC	CTCCGCTTCATAGAGGTAGT

**Table 2:** Univariate Cox regression analysis results.

Id	*z*	HR	HR.95L	HR.95H	*p* value
ACTA2	-2.382667536	0.7794916	0.635062344	0.956767726	0.017187709
MMP14	2.354059963	1.237687278	1.036344401	1.478147415	0.018569615
IGJ	-2.321172984	0.861330023	0.759325083	0.977037931	0.02027751
CTHRC1	2.184086222	1.176830023	1.016848758	1.361981212	0.028955913
CXCL12	-2.086278679	0.833517888	0.702454325	0.989035222	0.036953387
LAMB1	2.044172937	1.224712344	1.008385361	1.487447541	0.040936466
MFAP4	-1.973675604	0.871707401	0.760598958	0.999046587	0.048418641

**Table 3:** Multivariate cox regression analysis results.

Id	Coef	HR	HR.95L	HR.95H	*p* value
ACTA2	-0.379760522	0.684025199	0.531595384	0.880162783	0.003153906
IGJ	-0.152683995	0.858400938	0.751217636	0.980877092	0.024851869
CTHRC1	0.236265717	1.266510799	1.075666699	1.491214339	0.004578553
CXCL12	-0.153407609	0.857780012	0.69754989	1.054815661	0.145913422
LAMB1	0.313651044	1.368412138	1.104754211	1.694994017	0.004075463

**Table 4:** Independent prognostic univariate cox analysis results.

Variable	Coef	HR	HR.95 L	HR.95H	*p* value
riskScore	0.554622883	1.741284194	1.41049963	2.149642991	2.47E-07
STAGE	0.543228104	1.721555261	1.274933191	2.324633586	0.000392502
T	0.499131316	1.647289675	1.245504476	2.178686088	0.000467059
M	1.131809556	3.101263336	0.748842494	12.8436011	0.118512375
Age	0.016505353	1.016642319	0.993511015	1.040312175	0.15985152
Gender	-0.17646729	0.838226197	0.472510705	1.486999448	0.546261178
N	0.15060375	1.162535912	0.159530784	8.471654902	0.88185331
Grade	0.011591034	1.01165847	0.696375217	1.469685933	0.951493367

**Table 5:** Independent prognostic multivariate cox analysis results.

Id	Coef	HR	HR.95L	HR.95H	*p* value
STAGE	0.47806429	1.612949176	1.181104647	2.202688011	0.002639371
riskScore	0.493909346	1.638709999	1.323983571	2.028250591	5.65*E* − 06

## Data Availability

The TCGA dataset can freely be acquired from the TCGA database (https://portal.gdc.cancer.gov/), and the ICGC-HCC datasets can freely be downloaded from the ICGC database (http://dcc.icgc.org).

## Supplementary Materials

Supplementary 1 Table S1: 107 CAF-DEGs.

Supplementary 2 Table S2: 65 drugs that were different between the high-risk and low-risk groups.
