# Supplementary material for: Cancer Progression Mediated by CAFs Relating to HCC and Identification of Genetic Characteristics Influencing Prognosis
Source: J Oncol. 2022 Oct 15;2022:2495361. doi: 10.1155/2022/2495361 (PMC9590114; doi:10.1155/2022/2495361)
Supplement: Supplementary 1 — Table S1: 107 CAF-DEGs. [file 2495361.f1.docx]

Table S1 107 CAF-DEGs.

| NO. | Gene names | NO. | Gene names | NO. | Gene names | NO. | Gene names |
| --- | --- | --- | --- | --- | --- | --- | --- |
| 1 | ACTA2 | 28 | COMP | 55 | JAG2 | 82 | PECAM1 |
| 2 | AKR1C4 | 29 | CREB3L1 | 56 | JUNB | 83 | PLAU |
| 3 | ASPN | 30 | CTHRC1 | 57 | LAMB1 | 84 | PODN |
| 4 | AXL | 31 | CTSK | 58 | LBH | 85 | POSTN |
| 5 | BCL6B | 32 | CXCL12 | 59 | LTBP2 | 86 | PRELP |
| 6 | BIRC3 | 33 | CXCL14 | 60 | LTBP4 | 87 | PREX1 |
| 7 | CAV1 | 34 | CYP4F12 | 61 | LUM | 88 | PTGIS |
| 8 | CCDC3 | 35 | DDIT4 | 62 | LZTS1 | 89 | PTRF |
| 9 | CCDC80 | 36 | ECM1 | 63 | MAP1B | 90 | SFRP4 |
| 10 | CCL2 | 37 | EGR2 | 64 | MCAM | 91 | SFRP5 |
| 11 | CD163 | 38 | EHD2 | 65 | MFAP4 | 92 | SH2D3C |
| 12 | CD200 | 39 | EHD3 | 66 | MFGE8 | 93 | SMOC2 |
| 13 | CD248 | 40 | EMILIN1 | 67 | MMP14 | 94 | SOCS3 |
| 14 | CD34 | 41 | ESAM | 68 | MS4A6A | 95 | SPARC |
| 15 | CD4 | 42 | FGL2 | 69 | MS4A7 | 96 | SRGN |
| 16 | CD59 | 43 | FHOD1 | 70 | NGFR | 97 | SRPX |
| 17 | CD93 | 44 | FLNA | 71 | NOTCH3 | 98 | SULF1 |
| 18 | CEMIP | 45 | FRZB | 72 | NOV | 99 | TACSTD2 |
| 19 | CHST4 | 46 | GALNT18 | 73 | OLFML2A | 100 | TBX2 |
| 20 | COL1A1 | 47 | GPR56 | 74 | OLFML2B | 101 | TF |
| 21 | COL1A2 | 48 | HAND2 | 75 | OLFML3 | 102 | TMEM119 |
| 22 | COL4A1 | 49 | HEG1 | 76 | PALD1 | 103 | TPM4 |
| 23 | COL4A2 | 50 | HEYL | 77 | PAMR1 | 104 | TUBA1A |
| 24 | COL5A1 | 51 | IGJ | 78 | PAPLN | 105 | UNC5B |
| 25 | COL5A2 | 52 | ITGA3 | 79 | PCDH12 | 106 | VCAN |
| 26 | COL6A3 | 53 | ITGA9 | 80 | PDGFRA | 107 | VIM |
