# Supplementary material for: Cancer Progression Mediated by CAFs Relating to HCC and Identification of Genetic Characteristics Influencing Prognosis
Source: J Oncol. 2022 Oct 15;2022:2495361. doi: 10.1155/2022/2495361 (PMC9590114; doi:10.1155/2022/2495361)
Supplement: Supplementary 2 — Table S2: 65 drugs that were different between the high-risk and low-risk groups. [file 2495361.f2.docx]

Table S2. 65 drugs showed significant differences in the high and low risk groups

| Temsirolimus |
| --- |
| CI.1040 |
| NU.7441 |
| AZD8055 |
| AICAR |
| AMG.706 |
| DMOG |
| KU.55933 |
| Metformin |
| EHT.1864 |
| Dasatinib |
| NVP.BEZ235 |
| PD.0325901 |
| AZD.0530 |
| NVP.TAE684 |
| AKT.inhibitor.VIII |
| Vorinostat |
| GDC0941 |
| PD.173074 |
| Erlotinib |
| Docetaxel |
| WO2009093972 |
| Rapamycin |
| AZD6244 |
| JNJ.26854165 |
| BI.D1870 |
| MG.132 |
| BX.795 |
| A.770041 |
| PD.0332991 |
| Z.LLNle.CHO |
| AP.24534 |
| Parthenolide |
| GW.441756 |
| Nilotinib |
| OSI.906 |
| X17.AAG |
| GDC.0449 |
| AZD6482 |
| WH.4.023 |
| PF.4708671 |
| Axitinib |
| TW.37 |
| SB590885 |
| Thapsigargin |
| NSC.87877 |
| Cyclopamine |
| CMK |
| RDEA119 |
| Gefitinib |
| Sorafenib |
| CEP.701 |
| Imatinib |
| Methotrexate |
| ABT.263 |
| Vinblastine |
| AZD7762 |
| Lapatinib |
| AZ628 |
| GNF.2 |
| Bryostatin.1 |
| Camptothecin |
| Nutlin.3a |
| FH535 |
| ZM.447439 |
